# Photosynthetic responses of sun- and shade-grown barley leaves to high light: is the lower PSII connectivity in shade leaves associated with protection against excess of light?

**DOI:** 10.1007/s11120-014-9969-8

**Published:** 2014-01-21

**Authors:** Marek Zivcak, Marian Brestic, Hazem M. Kalaji, Govindjee

**Affiliations:** 1 Department of Plant Physiology, Slovak Agricultural University, Tr. A. Hlinku 2, 949 76 Nitra, Slovak Republic; 2 Department of Plant Physiology, Faculty of Agriculture and Biology, Warsaw Agricultural University SGGW, Nowoursynowska 159, 02-776 Warsaw, Poland; 3 Department of Plant Biology, University of Illinois at Urbana-Champaign, 265 Morrill Hall, 505 South Goodwin Avenue, Urbana, IL 61801 USA; 4 Department of Biochemistry and Center of Biophysics & Quantitative Biology, University of Illinois at Urbana-Champaign, 265 Morrill Hall, 505 South Goodwin Avenue, Urbana, IL 61801 USA

**Keywords:** Barley, Chlorophyll *a* fluorescence, Photoinhibition, Sun and shade leaves, Electron transport, PSII excitonic connectivity

## Abstract

In this study, we have compared photosynthetic performance of barley leaves (*Hordeum vulgare* L.) grown under sun and shade light regimes during their entire growth period, under field conditions. Analyses were based on measurements of both slow and fast chlorophyll (Chl) *a* fluorescence kinetics, gas exchange, pigment composition; and of light incident on leaves during their growth. Both the shade and the sun barley leaves had similar Chl *a*/*b* and Chl/carotenoid ratios. The fluorescence induction analyses uncovered major functional differences between the sun and the shade leaves: lower connectivity among Photosystem II (PSII), decreased number of electron carriers, and limitations in electron transport between PSII and PSI in the shade leaves; but only low differences in the size of PSII antenna. We discuss the possible protective role of low connectivity between PSII units in shade leaves in keeping the excitation pressure at a lower, physiologically more acceptable level under high light conditions.

**Electronic supplementary material:**

The online version of this article (doi:10.1007/s11120-014-9969-8) contains supplementary material, which is available to authorized users.

## Introduction

Plants live in varied environments and they are exposed to competition with others; further, they possess ability to adjust to different light conditions. However, they differ in their acclimation capacity to shade (Murchie and Horton 1997). Acclimation to different light intensities involves changes in the organization and/or abundance of protein complexes in the thylakoid membranes (Timperio et al. 2012). Leaves of pea plants grown in low light (LL) were found to have lower levels of Photosystem II (PSII), ATP synthase, cytochrome *b*/*f* (Cyt *b*/*f*) complex, and components of the Calvin–Benson cycle (especially ribulose-1,5-bisphosphate carboxylase/oxygenase, Rubisco), while the levels of major chlorophyll *a*/*b*-binding light-harvesting complexes (LHCII), associated with PSII, were increased (Leong and Anderson 1984a, b). In addition, leaves of plants grown in LL showed lower number of reaction centers (Chow and Anderson 1987), as well as decreased capacity for oxygen evolution, electron transport, and CO_2_ consumption and a lower ratio of chlorophyll *a* to chlorophyll *b* (Chl *a*/*b*) (Leong and Anderson 1984a, b). Ambient light intensity also modulates the content of the thylakoid components as well as PSII/PSI ratios (Leong and Anderson 1986), as was confirmed also by Bailey et al. (2001, 2004) in *Arabidopsis thaliana* plants grown in low and high intensity of light; they observed an increase in the number of PSII units in high light (HL) and an increase in the number of PSI units in LL. In addition to an increase in the amount of light-harvesting complexes (LHCII), a typically lower Chl*a/*Chl*b* ratio was observed. Further, differences have been observed in the thickness of mesophyll layer and in the number and structure of chloroplasts (Oguchi et al. 2003; Terashima et al. 2005). All these features reflected in a higher capacity for oxygen evolution, electron transport, and CO_2_ consumption in the sun plants. In addition, changes in pigment content and in the xanthophyll cycle, involved in thermal dissipation of excess light energy, have been shown to play a prominent role in plant photoprotection (Demmig-Adams and Adams 1992, 2006). As expected, these changes were found to be much lower in shade than in sun plants (Demmig-Adams and Adams 1992; Demmig-Adams et al. 1998; Long et al. 1994). Further, plants acclimated to LL showed reduced photorespiratory activity (Brestic et al. 1995; Muraoka et al. 2000).

Under HL conditions, plants must cope with excess light excitation energy that causes oxidative stress and photoinhibition (Powles 1984; Osmond 1994; Foyer and Noctor 2000). Photoinhibitory conditions occur when the capacity of light-independent (the so-called “dark”) processes, to utilize electrons produced by the primary photoreactions, is insufficient: such a situation creates excess excitation leading to reduction of the plastoquinone (PQ) pool and modification of the functioning of PSII electron acceptors (Kyle et al. 1984; Setlik et al. 1990; Vass 2012). HL activates strategies for photoprotection and repair of the photosynthetic apparatus from photoinhibition (Melis 1999; Demmig-Adams et al. 1998; Adir et al. 2003). This adaptation could be provided by plants at different levels of light conversion and energy flux through the electron transport chain.

In the present study, we have made photosynthesis measurements, accompanied by extensive measurements on chlorophyll *a* fluorescence (ChlF), and, then, we analyzed the latter to obtain detailed information on primary events and electron transport (see e.g., Papageorgiou and Govindjee 2004) in sun and shade barley leaves. Most of the earlier studies on sun and shade leaves had used mainly the saturation pulse analysis (Bradbury and Baker 1981; Schreiber 1986); in this work, however, we have included the analysis of polyphasic fast ChlF kinetics (Strasser et al. 1995) that has provided new information on differences in sun and shade leaves. The O–J–I–P transient [O being the minimal fluorescence (*F*_0_), J and I are inflections; and P is the peak, equivalent to *F*_m_], observed clearly when plotted on a logarithmic time scale, was analyzed. The *F*_0_ to *F*_m_ kinetics can be divided into three rise phases: O–J (0–2 ms), J–I (2–30 ms), and I–P (30–300 ms) (Neubauer and Schreiber 1987; Strasser and Govindjee 1991; Stirbet and Govindjee 2011). When using the phase amplitude modulation (PAM) technique (Schreiber 1986), fluorescence rise after a saturating pulse is observed as a simple spike. According to the widely accepted interpretation, first proposed by Duysens and Sweers (1963), the fluorescence rise from *F*_0_ to *F*_m_ reflects the reduction of Q_A_, the first PQ electron acceptor of PSII. On the basis of this simple model, more complex mathematical models have been built, including that for the analysis of OJIP transient (Strasser et al. 1995, 2004), well known as “the JIP-test.” In this test the major inflection points of the fast fluorescence induction curve are used for the calculation of various parameters characterizing the structure and photochemical activity of photosynthetic samples. Although there are some limitations due to the use of a number of approximations (cf. Stirbet and Govindjee 2011), practical use of the model has clearly demonstrated that it can explain and predict the performance of photosynthetic samples under several conditions, especially when it is used in parallel with other techniques (Stirbet and Govindjee 2012; Kalaji et al. 2012). The mathematical analysis of fast chlorophyll induction, if properly used, brings additional information and hence, it enables researchers to investigate more precisely the function of PSII and its responses to changes in environmental and growth conditions (Strasser et al. 2000, 2004; Force et al. 2003; Zivcak et al. 2008; Repkova et al. 2008; Goltsev et al. 2012; Kalaji et al. 2011, 2012; Brestic and Zivcak 2013). In addition to commonly used parameters or rate constants, fast chlorophyll fluorescence induction provides us additional information, such as excitonic connectivity among PSII units, as has been discussed in the past (see a review by Stirbet 2013). This connectivity is associated with the sigmoidicity of the initial phase of fast fluorescence transient (Joliot and Joliot 1964) and it plays an important role in mathematical models estimating the redox poise of PSII electron acceptors on the basis of chlorophyll fluorescence measurements (Lavergne and Trissl 1995; Kramer et al. 2004).

In this paper, we have examined the status of photosynthetic apparatus in mature barley plants grown in different light conditions. As a typical annual grass adapted to sunny habitats, barley can serve as an interesting model, as one can expect different acclimations to shade than in woody plants or sciophytic species. The main conclusion of our paper is based mostly on analyses of fast and slow chlorophyll fluorescence. Up to now, there has been a lack of studies combining the two ChlF techniques (PAM and directly measured fluorescence transient) in light acclimation studies; our current studies, using both methods, contribute to a better understanding of light acclimation process of barley plants grown under sun and shade conditions. We also discuss the differences in PSII connectivity observed in sun and shade barley leaves, and present some ideas about possible role of differences in excitation energy transfer for maintaining the redox poise of PSII electron acceptors under physiologically acceptable range.

## Materials and methods

### Plant material and experimental design

 Plants of spring barley (*Hordeum vulgare* L.), variety Kompakt, were grown in 10 liter plastic pots filled with humus soil substrate. We grew 45 plants per pot. Four pots were exposed to full sunlight during their entire growth period, whereas 4 pots were placed in shade, provided with a non-woven textile cover over them; this reduced the photosynthetic active radiation (PAR) to ~13 % of the sunlight. Each pot represents one replication; i.e., there were four replications per treatment.

From the central part of each pot, one healthy penultimate leaf with almost horizontal position of the leaf blade (corresponding to position of light sensor) was chosen for measurements, i.e., 4 leaves from each treatment (sun vs. shade) were used subsequently for all the analyses. Before the start of measurements, leaf development was observed and leaves were measured after the full length of leaf was achieved. All the measurements were completed within a few days under controlled conditions, in order to prevent changes due to leaf age. After each noninvasive measurement, plants were exposed to moderate light for recovery for at least 1 h; immediately after the last measurement, analysis of assimilation pigments was done from the same position of the same leaf.

### Determination of light conditions

Photosynthetic active radiation (PAR) sensor (Li-190SA, Li-COR, USA) was installed above the plants in the field to collect light intensity, and was connected to a data logger (Li-1400, Li-COR, USA). This enabled us to measure the PAR value, its maximum, and to calculate the total input and to obtain average values of PAR for each treatment during canopy development. The total PAR input of any leaf was calculated as a sum of incident PAR (in mols of photons per unit area per second) between the appearance of the leaf and the time of performing photosynthesis and fluorescence measurements and the HL treatment. The middle part of mature leaves of barley (which was measured) was almost in a horizontal position; hence, the measured values of PAR almost fully corresponded to light intensities incident on leaves.

### Measurement of photosynthetic parameters

 Barley plants were transferred to the laboratory for photosynthesis (CO_2_ fixation) measurements at different light intensities (to provide light response curve; see “Introduction” section), for rapid light curves of ChlF (see below), and for ChlF induction curves that provided information on the photochemical efficiency of PSII, among other parameters (see “Discussion” section, for details). “Results” section describes the protocol for studying the effect of HL. Measurements were done on fully expanded penultimate leaves.

1. *Light response curve of photosynthesis* was measured using CIRAS-2 gas analyzer (PP Systems, USA). CO_2_ concentration was fixed at ~370 μmol CO_2_ mol air^−1^; the sample temperature was 25 °C; PAR light intensities were 100, 300, 600, 900, and 1200 μmol photons m^−2^ s^−1^, given at an interval of 15 min for each light increment.
2. *Rapid light curves for fluorescence* were made as described by White and Critchley (1999). Parameters of modulated ChlF were measured using Mini-PAM Fluorimeter (Walz, Germany) with PAR intensity of 152, 246, 389, 554, 845, 1164, 1795, and 2629 μmol photons m^−2^ s^−1^ (internal halogen lamp). The measured and calculated parameters of ChlF are shown in Table 1.

**Table 1:** Measured and calculated chlorophyll fluorescence parameters

Parameters	Name and basic physiological interpretation
Measured or computed inputs for calculation of the key fluorescence parameters	
*F, F′*	Fluorescence emission from dark- or light-adapted leaf, respectively
*F*_0_	Minimum fluorescence from dark-adapted leaf (PSII centers open); *F*_0_ was not corrected for PSI fluorescence, and for the possible presence of reduced Q_B_ that could produce some reduced Q_A_ in darkness.
*F*_m_, *F*_m_ *′*	Maximum fluorescence from dark- or light-adapted leaf, respectively (PSII centers closed)
*F*_V_ = *F*_m_ *−* *F*_0_	Maximum variable fluorescence from dark-adapted leaf
*F*_0_ *′* = *F*_0_/[(*F*_V_/*F*_m_) + (*F*_0_/*F*_m_ *′*)]	Minimum fluorescence from light-adapted leaf^12^
*F*_s_ *′*	Steady-state fluorescence at any light level
*α* = *χ*/*(χ* + 76*)*	Absorbance of incident PAR (photosynthetic active radiation) by leaf^9^
*χ*	Total chlorophyll content (in μmol m^−2^)
Key chlorophyll fluorescence parameters derived from the saturation pulse analysis	
*F*_V_ */F*_m_ = *1* *−* *(F*_0_ */F*_m_ *)*	Estimated maximum quantum efficiency (yield) of PSII photochemistry^1,7,10^
*Φ*_PSII_ = *(F*_m_ *−* *F′)/F*_m_ *′*	Estimated effective quantum yield (efficiency) of PSII photochemistry at given PAR^5^
ETR = 0.5 × *α* × PAR × *Φ*_PSII_	Rate of linear electron transport in PSII at given photosynthetic active irradiance (PAR), assuming that there is equal partitioning of absorbed light between PSI and PSII (constant value 0.5)^4,5^
NPQ = *(F*_m_ *−* *F*_m_ *′)/F*_m_ *′*	Non-photochemical quenching^3,8^
qP = *(F*_m_ *′* *−* *F*_s_ *′)/(F*_m_ *′* − *F*_0_ *′)*	Coefficient of photochemical quenching based on the “puddle” model (i.e., unconnected PSII units)^2,4,6^
qL = qP × (*F*_0_/*F*_s_ *′*)	Coefficient of photochemical quenching based on the “lake” model (i.e., fully connected PSII units)^12^
qCU = (*F*_m_ *′* − *F*_s_ *′*)/((*p*/(1–*p*)) × (*F*_s_ − *F*_0_ *′*) + *F*_m_ *′* − *F*_0_ *′*)	Coefficient of photochemical quenching based on the “connected units model” model (intermediate model)^11,13^ parameter *p* is defined in Table 2.
*Φ*_NO_ = 1/[NPQ + 1 + qL(*F*_m_/*F*_0_ − 1)	Quantum yield of non-regulated energy dissipation in PSII^13^
*Φ*_NPQ_ = 1 *−* *Φ*_PSII_ *−* *Φ*_NO_	Quantum yield of pH-dependent energy dissipation in PSII^13^

Based on ^1^ Kitajima and Butler (1975); ^2^ Schreiber (1986); ^3^ Schreiber et al. (1988); ^4^ Björkman and Demmig (1987); ^5^ Genty et al. (1989); ^6^ Bilger and Björkman (1990); ^7^ Krause and Weis (1991); ^8^ Walters and Horton (1991); ^9^ Evans (1993); ^10^ Schreiber et al. (1995); ^11^ Lavergne and Trissl (1995); ^12^ Oxborough and Baker (1997); ^13^ Kramer et al. (2004)
3. *Protocol for studying the effect of HL* was as described below First, photochemical efficiency of PSII (Φ_PSII_) was calculated from fluorescence measurements in leaves after they were kept in dark for 30 min. This was followed by a 15-min exposure to 50 μmol photons m^−2^ s^−1^ of light. Thereafter, leaves were exposed for 1 h to 1,500 μmol photons m^−2^ s^−1^ (obtained from an external halogen lamp, 2050-HB, with a filter eliminating wavelengths of light above 710 nm). During this time, 4 saturation light flashes (16,000 μmol photons m^−2^ s^−1^) were applied every 15 min. After 1, 5, and 15 min of dark period recovery from HL, Φ_PSII_ (Butler 1978; Quick and Stitt 1989; Havaux et al. 1991) was obtained.
4. *ChlF induction curve* was measured using Handy-PEA fluorimeter (Hansatech Instruments Ltd., UK). First, we measured fluorescence transient from leaves kept in darkness for 30 min; this was our control. Then, we applied HL (see above); and fluorescence transient was measured 30 min after recovery from light. Fast fluorescence transients, thus obtained, were analyzed by the so-called “JIP test” (Strasser and Strasser 1995; Srivastava et al. 1999; Strasser et al. 2000, 2004, 2010; for the assumptions used, and pros and cons, see Stirbet and Govindjee 2011). The measured and calculated JIP parameters are described in Table 2.

**Table 2:** Measured and calculated parameters derived from fast fluorescence kinetics

Parameter	Name and basic physiological interpretation
Basic JIP-test parameters derived from the OJIP transient^2,3,4,6^	
*F*_t_	Fluorescence level at time *t*
*F*_m_ = *F*_P_	Maximum fluorescence (the measured “peak” *F*_*P*_ value)
*V*_t_ = (*F*_t_ − *F*_50 μs_)/(*F*_m_ − *F*_50 μs_)	Relative variable fluorescence at time *t,* (*V*_J_, *V*_I_ at 2, 30 ms)
Area	Area above the OJIP curve between *F*_0_ and F_m_ and the F_m_ asymptote
*S*_m_ = Area/(*F*_m_ − *F*_50 μs_)	Normalized area; proportional to the size of plastoquinone pool
Quantum yields and probabilities^2,3,5,6,7^	
*φ*_Po_ = *F*_V_ */F*_m_ = *1* − (*F*_50 μs_/*F*_m_)	Maximum quantum yield of primary PSII photochemistry
*ψ*_ET2o_ = 1 − *V*_J_	Probability with which a PSII trapped electron is transferred from reduced Q_A_ to Q_B_
*Ψ*_RE1o_ = 1 − *V*_I_	Probability with which a PSII trapped electron is transferred from reduced Q_A_ beyond PSI
Specific energy fluxes expressed per active PSII reaction center (RC)^2,3,5,7^	
ABS/RC = (d*V*/d*t*_o_/*V*_J_) × (1*/φ*_Po_)	Apparent antenna size of active PSII RC
TR/RC = d*V/*d*t*_o_/*V*_J_	Maximal trapping rate of absorbed photons in RC
ET/RC = (d*V*/d*t*_o_/*V*_J_) × (1 − *V*_J_)	Electron transport flux from reduced Q_A_ to Q_B_ in active RC
DI/RC = [d*V*/d*t*_o_/*V*_J_] × [1/(*F*_0_/*F*_m_)]	Effective dissipation of energy in active RC
Connectivity among PSII units^4,7,8^	
*W*_E_ = 1 − [(*F*_2ms_ − *F*_300 μs_)/(*F*_2ms_ − *F*_50 μs_)]^1/5^	Model-derived value of relative variable fluorescence at 100 μs calculated for unconnected PSII units
*W* = (*F*_100 μs_ − *F*_50 μs_)/(*F*_2ms_ − *F*_50μs_)	Relative variable fluorescence at 100 μs
*C* = (*W*_E_ − *W*)/[*V*_J_ × *W* × (1 − *W*_E_)]	Curvature constant of initial phase of the O–J curve
*p*_2G_ = C × [*F*_50μs_/(*F*_2ms_ − *F*_50μs_]	Overall grouping probability
*p* = [*p*_2G_ × (*F*_m_/*F*_50μs_ − 1)]/[1 + *p*_2G_ × (*F*_m_/*F*_50μs_ − 1)]	Connectivity parameter
*ω* = *p* × [(*F*_m_ − *F*_50μs_)/*F*_m_]	Probability of the connectivity among PSII units

Based on ^1^ Malkin and Kok (1966); ^2^ Strasser et al. (1995); ^3^ Strasser et al. (2000); ^4^ Strasser and Stirbet (2001); ^5^ Strasser et al. (2004); ^6^ Strasser et al. (2010); ^7^ Stirbet and Govindjee (2011); ^8^ Stirbet (2013)
5. *Determination of Chl a, b and carotenoid content* Segments of penultimate leaves of sun and shade plants were homogenized using sea sand, MgCO_3_, and 100 % acetone; and then extracted with 80 % acetone. After 2-min centrifugation at 2,500 rpm, absorbance of the solution was measured, by a UV–Vis spectrophotometer (Jenway, UK), at 470, 647, and 663 nm, with a correction for scattering, measured at 750 nm. The concentrations of Chl *a*, Chl *b*, and carotenoids (Car) per leaf area unit were determined, using the equations of Lichtenthaler (1987):
  $${\text{Chl }}a \, = \, \left( {12.25 \times A_{663} {-} \, 2.79 \times A_{647} } \right) \times D$$
  $${\text{Chl }}b \, = \, \left( {21.50 \times A_{647} {-} \, 5.10 \times A_{663} } \right) \times D$$
  $${\text{Car }} = \, \left[ {\left( {1,000 \times A_{470} {-} \, 1.82 \times \left( {Chl \, a} \right) \, {-} \, 85.02 \times \left( {Chl \, b} \right)} \right)/198} \right] \times D$$
  Here, the concentrations of the pigments are calculated in mg dm^−3^; *A*_*n*_ is the absorbance at a given wavelength (*n*) after correction for scattering at 750 nm; *D* is the optical thickness of the cuvette; results were also recalculated in mg m^−2^ using the volume of solution and the area of leaf segments.

### Data analysis

All the experiments were conducted with four independent biological replicates. The differences between sun- and shade-grown leaves, as well as the effects of HL treatment on leaves differing in light acclimation, were analyzed by one-way analysis of variance (ANOVA) using software Statistica 9 (Statsoft Inc., Tulsa, OK, USA) for each parameter. Once a significant difference was detected, post-hoc Duncan’s multiple range tests at *P* < 0.05 were used to identify the statistically significant differences. Results shown in graphs and tables are presented as the mean value of four replicates ± standard error; in the tables, statistically significant differences are indicated by unequal small letters next to the values.

## Results

The results of measurements of PAR at the leaf level show 8 times higher average and 5 times higher maximum values incident on the sun leaves compared to those in the shade leaves. The PAR input, calculated as a total sum of incident PAR on the penultimate leaf (the second leaf below the spike, usually the largest one) from the time leaf was formed till it reached its maximum length, was 3.5 times higher for barley leaves in the sun than in the shade (see Table 1 of Supplementary Material, labeled as Suppl. Table 1); our data show slower leaf development under LL conditions. Shade leaves showed a lower photosynthetic pigment concentration and a higher leaf area than those grown under the sun. However, no significant changes were observed in the Chl*a*/Chl*b* and the Chl/carotenoid ratios (Table 3).

**Table 3:** The content of chlorophylls and carotenoids, the ratios of pigments, and the leaf area of the observed penultimate sun and shade leaves

Light regime	Content (mg m^−2^)			Chl *a/b* ratio	Chl/Car ratio	Leaf area (cm^2^)
	Chlorophyll *a*	Chlorophyll *b*	Carotenoids			
Sun	308.7 ± 1.8^a^	132.3 ± 5.2^a^	81.1 ± 1.7^a^	2.34 ± 0.1^a^	5.44 ± 0.2^a^	11.5 ± 1.4^a^
Shade	246.3 ± 7.2^b^	101.1 ± 8.6^b^	65.4 ± 2.0^b^	2.45 ± 0.2^a^	5.32 ± 0.4^a^	19.6 ± 2.4^b^

Sun—full light; shade—light level ~13 % of full light. Mean values ± SE from 4 replicates are presented. Letters indicate significant differences at *P* < 0.05 according to Duncan’s multiple range tests

### Photosynthesis and fluorescence characteristics before leaves were exposed to HL

Leaves from plants grown in LL regime showed saturation of photosynthesis at ~600 μmol photons m^−2^ s^−1^, while leaves from plants grown in full sunlight showed saturation of photosynthesis at ~1,200 μmol photons m^−2^ s^−1^; furthermore, the sun leaves had maximum CO_2_ assimilation rate of ~20 μmol CO_2_ m^−2^ s^−1^, almost two times higher than the shade leaves (~11 μmol CO_2_ m^−2^ s^−1^, Suppl. Fig. 1). This difference was not caused by stomatal effect; since at HL the CO_2_ content inside the shade leaves was higher than in the sun leaves, as indicated by the ratio of intercellular to atmospheric CO_2_ content (*Ci/Ca* ratio).

**Fig. 1:**
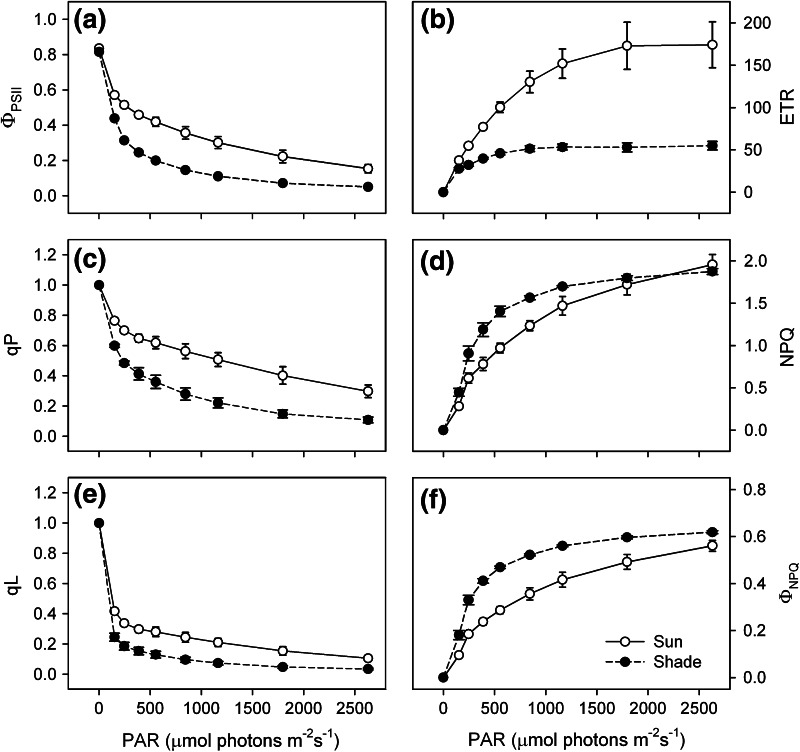
Chlorophyll *a* fluorescence parameters derived from the rapid light curves (at 0, 152, 246, 389, 554, 845, 1164, 1795, and 2629 μmol photons m^−2^ s^−1^, 15 s). **a** The photochemical efficiency of PSII (Φ_PSII_), **b** electron transport rate (ETR, inferred from fluorescence measurements after correction for different leaf absorbances, and assuming that PSII:PSI ratio is 1:1; Genty et al. 1989). **c** Photochemical quenching (qP) based on the “puddle” model (connectivity parameter (*p*) between different PSIIs = zero). **d** Non-photochemical quenching (NPQ), **e** photochemical quenching (qP) based on the “lake” model [connectivity parameter (*p*) between PSII units = 1]. **f** Quantum yield of non-photochemical quenching (Φ_NPQ_). Measurements were performed on penultimate leaves of spring barley plants acclimated to different light intensities (*open circle* sun leaf—100 % of daylight, *filled circle* shade leaf—13 % of daylight, their entire growth period). Mean values ± SE from 4 replicates

Results obtained by the so-called *rapid light curves* (see “Materials and methods” section) show that, with incremental increase of intensity in light flashes (up to about 1,150 μmol photons m^−2^ s^−1^), the photochemical efficiency of PSII (Φ_PSII_) and the photochemical quenching of ChlF (i.e., qP and qL) decreased gradually (Fig. 1): sun plants had higher values (about twofold) than in those kept in the shade (for definition of individual ChlF parameters see Tables 1, 2). Significant rise of electron transport rate (ETR) across PSII, as calculated from fluorescence data, was found in plants grown under HL (up to ~1,800 μmol photons m^−2^ s^−1^), while it was very low in the case of shade plants and did not change at higher light intensities (Fig. 1b). In these plants, thermal dissipation of excitation energy, as expressed by non-photochemical quenching of ChlF (NPQ) and of quantum yield of non-photochemical quenching (Φ_NPQ_), showed similar trends to that shown by calculated ETR, but more energy was dissipated as heat between ~390 and ~1,160 μmol photons m^−2^ s^−1^ of light intensity (Fig. 1d, f). Data shown in subfigures a, c, and e of Fig. 1 will be discussed later.

**Fig. 2:**
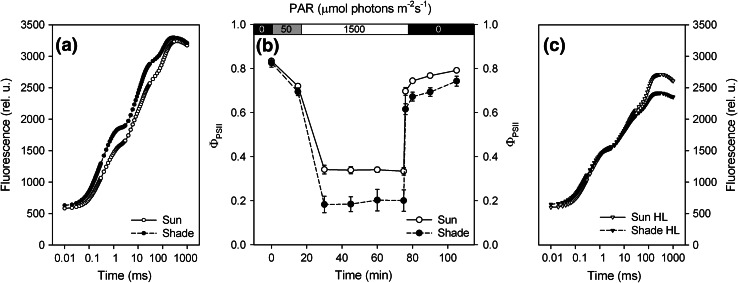
**a** Chlorophyll *a* fluorescence induction curves at 3,500 μmol photons m^−2^ s^−1^ of continuous red light up to 1 s for the sun and the shade leaves. Dark adaptation was for 30 min (for other details, see the legend of Fig. 1). **b** Photochemical efficiency of PSII (Φ_PSII_) with time, during the following protocol: 15 min of low light (50 μmol photons m^−2^ s^−1^), followed by high light (1,500 μmol photons m^−2^ s^−1^) for 1 h, and then 30 min of darkness. **c** Fluorescence induction curves at 3,500 μmol photons m^−2^ s^−1^ of continuous red light for 1 s recorded after 30-min recovery in the dark. (*open circle* sun leaf (100 % of daylight), *filled circle* shade leaf (13 % of daylight)). Mean values ± SE from 4 replicates

In shade plants, compared to sun plants, fast ChlF induction curve (the OJIP curve; see reviews: Stirbet and Govindjee 2011, 2012) showed no significant differences in *F*_0_ and *F*_m_ values and hence, the maximum quantum yield of PSII photochemistry Φ_Po_ was almost unaffected by the leaf ambient light environment. However, the shape of fast ChlF induction (Fig. 2a) was not identical in sun and shade leaves suggesting possible differences in energy fluxes at the donor as well as at the acceptor side of PSII (Strasser et al. 2000); this conclusion is supported by the calculated ChlF parameters (Table 4).

**Table 4:** Selected parameters derived from fast fluorescence kinetic measurements in the sun and the shade barley leaves before (B) and after they were exposed to high light (HL)

	Sun		Shade	
	B	HL	B	HL
*F*_0_	535 ± 8^a^	564 ± 4^b^	573 ± 21^b^	618 ± 9^c^
*F*_m_	3,233 ± 29^a^	2,710 ± 42^b^	3,294 ± 93^a^	2,416 ± 69^c^
*F*_V_/*F*_m_	0.84 ± 0.001^a^	0.79 ± 0.003^b^	0.83 ± 0.007^a^	0.74 ± 0.009^c^
*S*_m_	31.2 ± 2.9^a^	28.5 ± 1.2^a^	19.6 ± 0.8^b^	21.2 ± 1.6^b^
ψ_ET2o_	0.63 ± 0.01^a^	0.57 ± 0.01^ab^	0.55 ± 0.01^ab^	0.53 ± 0.01^b^
ψ_RE1o_	0.26 ± 0.01^a^	0.28 ± 0.01^a^	0.16 ± 0.003^c^	0.21 ± 0.01^b^
ABS/RC	2.22 ± 0.06^a^	2.30 ± 0.03^a^	2.58 ± 0.22^ab^	2.80 ± 0.13^b^
*p*_2G_	0.27 ± 0.05^a^	0.26 ± 0.04^a^	0.12 ± 0.03^b^	0.18 ± 0.02^ab^
*p*	0.51 ± 0.04^a^	0.45 ± 0.04^a^	0.28 ± 0.07^b^	0.29 ± 0.02^b^
*ω*	0.64 ± 0.05^a^	0.59 ± 0.05^a^	0.36 ± 0.09^b^	0.43 ± 0.03^b^

More detailed description and calculations are given in Tables 1 and 2 and their legends. Values represent the mean ± SE (*n* = 4). Letters indicate significant differences at *P* < 0.05 according to Duncan’s multiple range tests

Sun—full light; shade—light level ~13 % of full light. B—measurements before high light protocol; HL—measurements after high light protocol and dark adaptation (HL). Parameters: *F*_0_—minimum fluorescence in dark-adapted leaves; *F*_m_—maximum fluorescence in dark-adapted leaves; *F*_V_/*F*_m_—related to maximum photochemical efficiency of PSII; *S*_m_—normalized area; ψ_ET2o_—probability with which trapped electron is passed beyond Q_A_; ψ_RE1o_—probability with which trapped electron is passed beyond PS I; *p*_2G_—overall grouping probability of PSII units; *p*—connectivity parameter; *ω*—probability of connectivity among PSII units

The first part of fast ChlF kinetics (from 0.05 to 2 ms) measured at high frequency (up to 100 kHz) was used to estimate the connectivity parameter among PSII units (Joliot and Joliot 1964; Strasser and Stirbet 2001; Joliot and Joliot 2003; Stirbet 2013). Calculated values of parameters associated with connectivity, the curvature parameter—C and probability of connectivity among PSII units—*p* (as defined by Strasser and Stirbet 2001), were ~2 times higher in sun leaves compared to those in the shade (Table 4). This connectivity reflects the fact that the light-harvesting antenna is not associated with only one separated RC, as assumed in many models, including the JIP test (cf. Stirbet and Govindjee 2011), but that the RCs are partially connected (Butler 1978; Lavergne and Trissl 1995; Kramer et al. 2004), meaning that the excitation energy of closed RCs can be transferred to a number of nearby open RCs. This calculation was based on deviations of basic hyperbolic shape of the initial part of the O–J phase (Strasser and Stirbet 2001). The initial slope of variable fluorescence within rapid ChF kinetics indicated more rapid initial accumulation of closed RCs in the shade compared to the sun plants (cf. Strasser et al. 2004). Moreover, the higher values of ChlF at the J and the I steps, and hence higher *V*_J_ and *V*_I_ values in the shade plants point to limited number of electron carriers on the PSII acceptor side (Lazar 1999, 2006). Detailed analysis, based on the selected parameters (Table 4) in shade leaves, suggest a decreased size of the pool of PSII and PSI electron carriers (from Q_A_ to ferredoxin) (parameter normalized Area, *S*_m_), as well as a decrease in the number of Q_A_ turnovers between *F*_0_ and *F*_m_ and hence a decreased number of electron carriers. These results are supported also by calculated values of the probability of electron transport from reduced Q_A_ to Q_B_ (ψ_ET2o_), as well as of the probability ψ_ET2o_, which expresses the fraction of PSII trapped electrons that are transferred further than Q_A_ in the electron transfer chain. The probability of electron transport from the PSII to the PSI acceptor side (ψ_RE1o_), estimated as 1—*V*_I_ (see Table 2), was higher in the sun than in the shade leaves. The difference of the probabilities of electron transport to the PSI acceptor side (ψ_RE1o_) between sun and shade leaves was relatively much higher than that corresponding to ψ_ET2o_ indicating a major limitation of electron transport between Q_B_ and the PSI electron acceptors in the shade leaves.

#### Characteristics of the photosynthesis apparatus after HL treatment

During 15 min of exposure to LL intensity (50 μmol photons m^−2^ s^−1^), which gave minimal photosynthesis, the photochemical efficiency of PSII (Φ_PSII_) was the same in the sun and the shade leaves. Fifteen minutes after the application of HL (1,500 μmol photons m^−2^ s^−1^), Φ_PSII_ in the shade leaves dropped almost to half the value to those in the sun leaves (Fig. 2b). However, during the HL treatment the quantum yield and hence the ETRs slightly increased in the shade leaves and the difference between the sun and shade leaves after 1 h of HL had diminished.

#### Characteristics of photosynthesis and fluorescence during recovery from HL treatment

After HL treatment, photochemical efficiency of PSII (Φ_PSII_) recovered when leaves from the shade plants were transferred to dark; during the recovery, Φ_PSII_ increased gradually. However, leaves from the sun plants had higher values of Φ_PSII_ than those from the shade plants (Fig. 2b).

The variable ChlF after 30 min of dark relaxation was not fully relaxed (see Fig. 2c). This seems to be the most pronounced effect on ChlF when compared to its status before the light treatment (Fig. 2a). Moreover, the difference between the sun and the shade leaf indicated that the level of photoinhibition was slightly higher in the shade plants. Based on Fm values, before and after HL treatment (Fig. 2), the non-relaxed fraction of quantum yield after 30 min in dark (*q*_i_) was 0.30 ± 0.04 in the sun leaves and 0.39 ± 0.07 in the shade leaves.

Increase of relative variable fluorescence at 2 ms (*V*_J_) indicates stronger limitation of electron transport from Q_A_ to Q_B_ as shown also numerically by the values of probability (ψ_ET2o_) of trapped PSII electron transfer from reduced Q_A_ to Q_B_ (Table 4).

The variable Chl fluorescence increase from I to P represents the measure of electron transport from Q_B_ beyond PSI (Munday and Govindjee 1969; Schansker et al. 2003). As is evident by the values of the probability with which the electron moves toward PSI end acceptors, ψ_RE1o_, the electron transport between PSII and PSI after HL treatment becomes less limited (Table 4), especially in shade leaves. (For a detailed discussion on the interpretation of the J–I–P rise (the so-called thermal phase of fast ChlF kinetics), see a review by Stirbet and Govindjee 2012).

Another explanation for the above results is that HL treatment affects the post-illumination redox state of the PQ pool, and the activation state of the PS I acceptor side (e.g., due to FNR activity) probably does not decay within the 30-min dark period that was used before the measurements. Stromal components can donate electrons to the PQ pool in the dark. Reduction in the dark can be substantially stimulated by pre-illumination with strong light (Asada et al. 1992). An increase of PQ-pool reduction with respect to the control will induce an increase of the J-step (Toth et al. 2007) and, hence, of all the parameters based on the values of *V*_J_. This is also supported by increased values of *F*_0_ in samples 30 min after HL treatment.

The changes of connectivity parameters (*p*_2G_, *p*, *ω*) after HL treatment were mostly insignificant (Table 4); moreover, according to Laisk and Oja (2013), estimates of *p* parameter can be strongly influenced by the redox status of the PQ pool. Since *F*_0_ value may increase in samples after HL treatment, calculated values of connectivity parameters may not be used as a measure of true PSII connectivity. Nevertheless, the insignificant differences between the *F*_0_ values before and after HL treatment and the maintained significance of differences between the sun and shade leaves suggest that the estimate of connectivity parameters could not be as prone to errors due to PQ redox status as expected.

The membrane model parameters (Table 4) show energy flux parameters per active RC. A higher value of the inferred absorbance per RC (ABS/RC) in shade leaves before HL treatment (~2.6) as compared to the sun leaves (~2.2) seems to indicate increased antenna size per active RC (Strasser et al. 2000; Stirbet and Govindjee 2011). However, a correction for connectivity (Suppl. Table 2; see information given in parentheses), i.e., multiplying the ABS/RC by 1 + *C* where *C* is the curvature constant of the relative variable fluorescence curve (Force et al. 2003), eliminated the difference in antenna size between the sun (~3.1) and the shade leaves (~3.1), as the connectivity before HL treatment was found to be substantially higher in sun leaves (Table 4).

## Discussion

As shown under Results, the penultimate leaf (the second leaf below the spike, usually the largest one) in shade-grown plants fulfilled the major conditions for it to be called “shade leaf” (Lichtenthaler et al. 1981; Givnish 1988). Although the total Chl content was lower per leaf area in the shade leaves, the Chl*a*/Chl*b* ratio was statistically similar in leaves grown at different light intensities. However, it is well known (Lichtenthaler 1985; Evans 1996) that under conditions of HL, for example, under a sunny habitat, plants have usually smaller PSII antenna size. On the other hand, under low-light conditions, in a shady habitat, plants have larger PSII antenna size; here usually the amount of the outermost PSII antenna proteins (the major peripheral antenna proteins) change in response to light conditions, while the other PSII antenna proteins, that is, the core antenna proteins and the inner peripheral antenna proteins (the minor peripheral proteins), remain unchanged (Anderson et al. 1997; Tanaka and Tanaka 2000). Hence, the lower value of Chl*a*/Chl*b* ratio is expected in shade leaves, as has been documented in many studies, e.g., in the sun and the shade leaves of forest trees (Lichtenthaler et al. 2007).

Our results on the absence of difference in Chl*a*/Chl*b* ratio between HL and LL grown plants (Table 3) confirm the results of Falbel et al. (1996), also in barley leaves; Kurasova et al. (2003) and Krol et al. (1999) had also observed relatively low differences. This seems to be consistent with the size of PSII antenna estimated by corrected values of ABS/RC for connectivity (see “Results” section). Hence, both pigment composition and fast ChlF induction analysis indicate that barley belongs to a group of plants with fixed antenna size (Tanaka and Tanaka 2000). Further, Murchie and Horton (1997) had found similar results on other shade-grown plants, where the Chl content had decreased but there was no change in the Chl*a*/Chl*b* ratio. Thus, we conclude that the decrease of Chl*a/*Chl*b* ratio in LL is not a universal phenomenon, and the level of its dependence on light intensity strongly depends on plant species.

In contrast to results on the antenna size, the electron transport chain was strongly affected by the light levels under which plants were grown. Our data on the analysis of the fast ChlF induction (Strasser et al. 2000, 2004, 2010) show that the parameters attributed to the probability of electron transfer from the reduced Q_A_ to Q_B_ (ψ_ET2o_) and the probability of electron transfer from Q_A_ to beyond the PSI (ψ_RE1o_) were higher in the sun than in the shade leaves (0.63 vs. 0.55 for ψ_ET2o_; 0.26 vs. 0.16 for ψ_RE1o_). This conclusion needs to be confirmed by measuring electron transport in PSI (P700). However, the above inference is suggested to be related to the limited pool size of electron acceptors, as indicated by measurements on the normalized area over the ChlF curve (*S*_m_), related to the number of electron carriers (*N*), which indicate a decrease of the pool of electron carriers by almost 27 %. These observations support the findings of Cascio et al. (2010) who found that one of the most important differences between ChlF transient in the sun and the shade leaf is a higher relative variable fluorescence at 30 ms (*V*_I_).

**Fig. 3:**
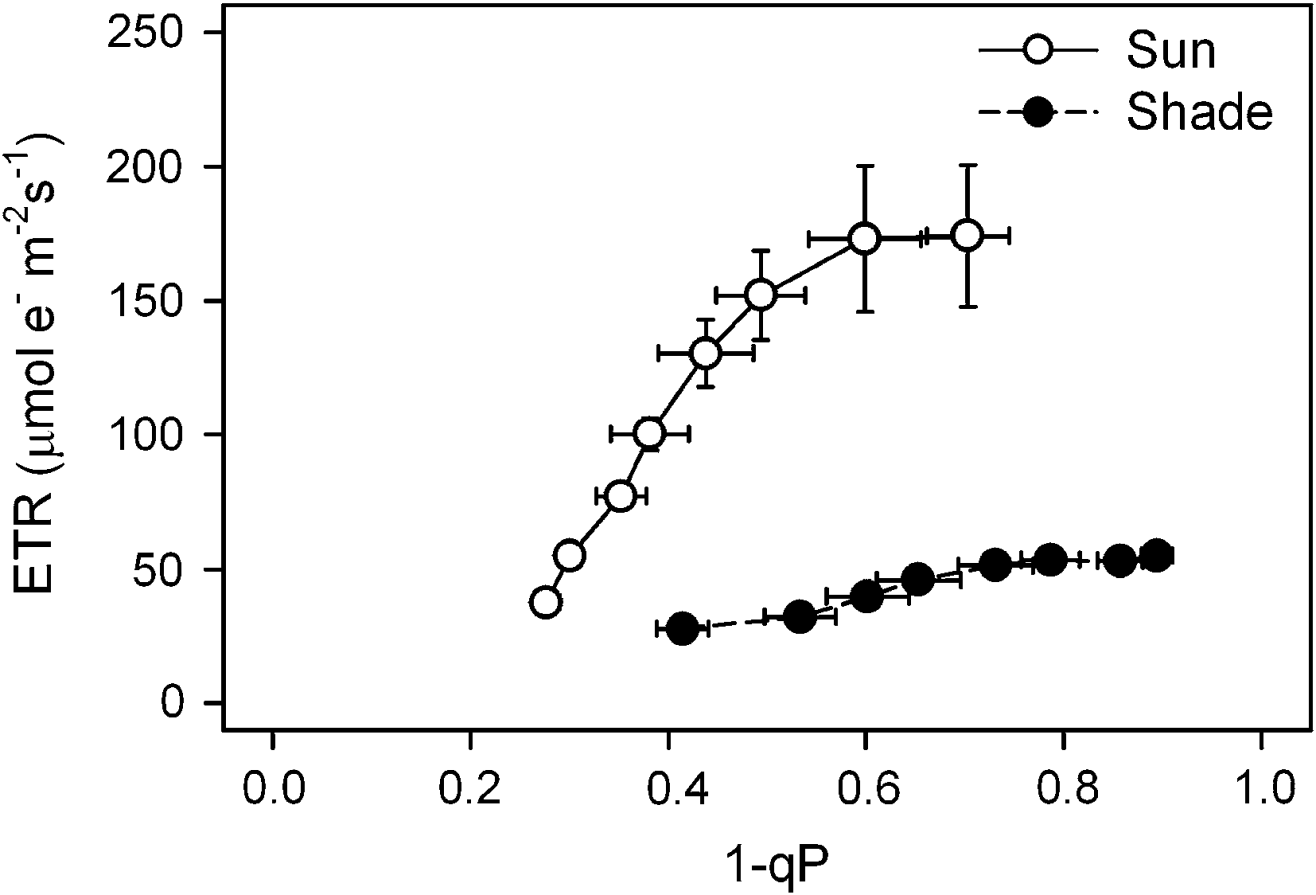
Relation of the calculated electron transport rate (ETR) and the approximate redox state of Q_A_ (1-qP), where the qP represents the coefficient of photochemical quenching. Chlorophyll *a* fluorescence parameters were derived from the rapid light curves (see Fig. 1)

The final I–P part of the fast ChlF transient (and the related ψ_RE1o_) reflects the rate of reduction of ferredoxin (Schansker et al. 2003, 2005) and it is taken as a measure of relative abundance of PSI with respect to PSII (Desotgiu et al. 2010; Cascio et al. 2010; Bussotti et al. 2011). For a complete discussion on the J to P phase, see Stirbet and Govindjee (2012). On the other hand, a limitation can also be caused by other components of electron transport between PSII and end PSI acceptors. Many studies have shown that Cyt *b6*/*f* may be the site of the rate-limiting step in the electron transport (Stiehl and Witt 1969; Haehnel 1984; Heber et al. 1988; Eichelmann et al. 2000). Golding and Johnson (2003) have described regulation of electron transport through Cyt*b6*/*f*; they documented this phenomenon by measurement of the PSI reaction center absorbance change, measured at 700 nm (P700). The rate limitation in the electron transport may be examined through the relationship between the redox poise of PSII electron acceptors and the ETR (Rosenqvist 2001), as shown in Fig. 3. The value of (1-qP) representing the approximate redox state of Q_A_, i.e., the Q_A_^−^/Q_A_ (total) (Schreiber and Bilger 1987; Weis et al. 1987) or excitation pressure (Ögren and Rosenqvist 1992), as used by Rosenqvist (2001), increased with light intensity. Similarly, the ETR was expected to grow in direct proportion to excitation pressure. However, while the relationship between the value of excitation pressure and ETR in sun leaves show an almost linear and a steep increase, we observed only a slight increase due to very low ETR, even at HL (ETR and qP values are shown in Fig. 1), in the shade leaves. This supports the conclusion from fast ChlF kinetics, which indicates a severe limitation in the electron transport of the shade barley leaves compared to the sun barley leaves. Rosenqvist (2001) has presented similar differences in the sun and the shade leaves of *Chrysanthemum*, *Hibiscus*, and *Spathiphyllum.*

Consistent with the above results, a substantial difference between ETR/(1-qP) ratio was found between light-adapted sun and shade barley leaves during photoinhibitory treatment (data not shown here). The high excitation pressure is considered to be directly related to the photoinhibitory damage (Ögren 1991; Ögren and Rosenqvist 1992; Gray et al. 1996; Kornyeyev et al. 2010); however, the level of photoinhibition is inversely proportional to the level of photoprotection and to the ability to repair photodamaged PSII elements. Many studies show that both the photoprotection and the repair ability increase with longtime exposure to high excitation pressure, mostly at HL intensities (Tyystjärvi et al. 1992; Niinemets and Kull 2001). Together with a very low ETR and non-photochemical quenching (of Chl fluorescence), similar to that in sun plants, we could expect severe photoinhibitory damage in shade plants exposed to HL treatment. However, low differences in photoinhibitory effects (*q*_I_) between sun and shade leaves did not correspond with high differences in excitation pressure. One possible explanation is that the values of the excitation pressure may have been estimated inaccurately and 1-qP values are really not the true estimates of the PSII redox poise. Rosenqvist (2001) has discussed the possible “inaccuracy” of the calculated values of photochemical quenching, qP, as it probably inaccurately estimates the fraction of oxidized Q_A_ due to “connectivity among PSII units” (Joliot and Joliot 1964; Paillotin 1976; Joliot and Joliot 2003).

The concept of connectivity among PSII units is included in many models; however, there is still a lack of reliable data for the correct values of probability parameter *p* in different plant species. Kramer et al. (2004), based on the data published by Lazar (1999), have reported that the *p* value in higher plants is usually higher than 0.6 (supported by Joliot and Joliot 2003, who obtained *p* = 0.7); in such a case, the qL would reflect fully the redox state of Q_A_. On the other hand, the data published by Kroon (1994) show *p* values between 0.25 and 0.45. Further, Strasser and Stirbet (2001), using direct measurements of fast ChlF kinetics, found a value of *p*_2G_ around 0.25, using both ChlF curves in the presence and the absence of DCMU; it represents a *p* value of ~0.5 (Stirbet 2013). Although the connectivity is estimated from the initial part of chlorophyll fluorescence curve, it does not mean that it is valid only for the initial phase. According to the theory of PSII connectivity, the migration possibilities for excitons that are inferred from the sigmoidal shape of fluorescence induction also influence the efficiency of utilization of absorbed light for trapping electrons in the RC and hence, it has an effect on the entire fluorescence kinetics (Lavergne and Trissl 1995). Recently, Tsimilli-Michael and Strasser (2013) documented that the *p*_2G_ can be correctly calculated even if only some of the RCs are inactive as well as in the case when the true *F*_m_ (all RCs closed) is not reached experimentally. Using the method of Strasser and Stirbet (2001), we have calculated values of *p*_2G_, *p*, and *ω* for both the sun and the shade barley leaves (Table 4), obtaining similar values, as previously mentioned, in sun leaves (*p*_2G_ ~ 0.27, *p* ~ 0.51, *ω* ~ 0.64), but substantially lower values in shade leaves (*p*_2G_ ~ 0.12, *p* ~ 0.28, *ω* ~ 0.36).

As the connectivity parameter (*p*) plays an important role in the calculation of many parameters estimating the redox state of Q_A_, we have compared the estimates based on three different models, as mentioned above: (1) The “Puddle” or “separate units” model; here qP is related to the redox state of Q_A_, and *p* = 0 (Krause et al. 1982; Bradbury and Baker 1984; Quick and Horton 1984; Schreiber et al. 1986). (2) The “Lake” model, where PSII units are fully connected with each other, and the open reaction centers compete for all the available excitons, and *p* = 1 (Kramer et al. 2004). (3) The “connected unit” model, where connectivity parameter *p* ranges between 0 and 1 (Joliot and Joliot 1964). In the model of Lavergne and Trissl (1995), each RC possesses its own antenna (like the “Puddle” model), but with a defined probability for transfer of excitation energy from one antenna system to another, similar to the “Lake” model (Kramer et al. 2004). By substituting *p* values obtained from fluorescence induction data into equations, we have calculated qCU (connected units) parameter in analogy to qP, which takes into account the degree of PSII connectivity (Lavergne and Trissl 1995; Kramer et al. 2004). Then we expressed the excitation pressure, representing the reduction of primary PSII electron acceptor (Q_A_^−^/Q_A total_), calculated using the “Puddle” model for the unconnected PSII units (parameter: 1-qP); as well as two more parameters: (i) (1-qCU) for the “connected units” model and (ii) (1-qL) for the “Lake” model.

**Fig. 4:**
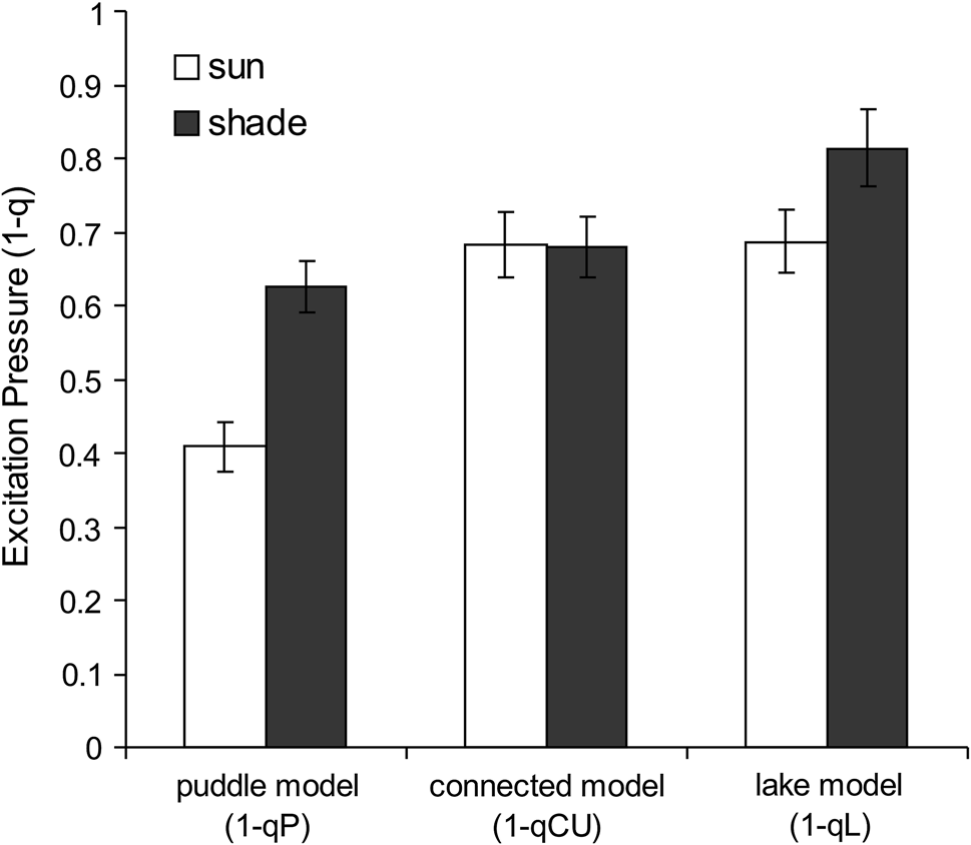
The excitation pressure, representing the reduction status of primary PSII electron acceptor (Q_A_^−^/Q_A tot_) calculated using the “puddle” model for unconnected PSII units (parameter 1-qP), the connected model according to Lavergne and Trissl (1995) using parameter 1-qCU, and “Lake” model (parameters 1-qL). The data of measurements done after 15 min in high light (1,500 μmol photons m^−2^ s^−1^) are shown. Parameters qP and qCU and qL represent photochemical quenching, the fraction of open PSII reaction centers calculated according to “puddle” (qP), “connected units” (qCU), and “Lake” (qL) models (see Table 1)

The estimate of Q_A_ reduction (Q_A_^−^/Q_A total_) at HL (1,500 μmol photons m^−2^ s^−1^) in the sun and shade leaves of barley, by parameters derived from “Puddle” (1-qP) or “Lake” (1-qL) model (Fig. 4), shows substantially higher excitation pressure in shade leaves than in sun leaves, as a consequence of low electron transport in shade leaves. As we can prejudge neither the higher photoprotection capacity (as shown by the parameter NPQ, Fig. 1) nor the capacity for the repair of photodamaged PSII components (as mentioned earlier), we can expect substantially higher levels of photoinhibition in shade leaves compared to the sun leaves. In contrast to the expectations for the shade-grown barley leaves, we observed only a small difference in the photoinhibitory level in these leaves, compared to the sun-grown leaves, as shown by the dark relaxation kinetics of variable Chl fluorescence (Fig. 2b) or fast ChlF kinetics (Fig. 2c). One of the possible explanations is that the difference in excitation pressure was not as pronounced as indicated by the 1-qP or the 1-qL parameters. Further, the estimate of excitation pressure, based on the “connected unit” model (1-qCU) that takes into account different values of PSII connectivity in the sun and shade leaves, indicates much lower differences in Q_A_ redox state between the sun and the shade leaves (Fig. 4). These results suggest that in the shade leaves, excitation energy is transferred from antenna into RCs much less efficiently, and hence, fewer electrons get into the intersystem chain, and this results in minor photoinhibitory damage.

Strasser et al. (2000) have suggested that connectivity may represent a tool by which the photosynthetic apparatus may regulate the use of excitation energy to adapt to new conditions. This is supported by results on PSII connectivity, shown mostly as the so-called L-band (around 0.1 ms) observed if the differences between relative variable fluorescence (*V*_t_) of two samples are plotted (not shown here). The appearance of L-bands indicates changes in the curvature of the initial phase of ChlF (Strasser et al. 2000), influenced, e.g., by drought (Oukarroum et al. 2007; Redillas et al. 2011), aluminum toxicity (Jiang et al. 2008), and high temperature (Brestic et al. 2012). In this respect, the changes in connectivity may represent the outward manifestation of adjustment of the PSII structure under environmental stress.

However, there is a lack of experimental results confirming the effects directly related to PSII connectivity. The issue of connectivity as well as methods of its estimate are still under discussion. Vredenberg (2008) reported much lower connectivity in dark-adapted chloroplasts than was estimated by sigmoidicity of fluorescence curve in the presence of DCMU. He also found that the sigmoidicity can also be described by two sequential, not parallel, exponential processes; this was confirmed by experimental results of Schansker et al. (2011). However, Laisk and Oja (2013), unlike their previous paper challenging the role of PSII connectivity (Oja and Laisk 2012), documented that fluorescence induction curve in the presence of DCMU was well fitted by a model assuming the PSII antenna to be excitonically connected in domains of four PSII. However, they are inclined to the view that the connectivity is constant and the apparent variability in PSII connectivity reflects the fact that one usually neglects the pre-reduction of PSII acceptor side carriers. Schansker et al. (2013), however, suggest separating the results obtained in the presence of DCMU from the sigmoidicity observed in the absence of DCMU, since the results, mentioned above, do not necessarily imply that connectivity between PSII antenna does not exist. In addition to fluorescence-based results, supporting the existence of connectivity among PSII units (Joliot and Joliot 1964; Briantais et al. 1972; Paillotin 1976; Moya et al. 1977; Malkin et al. 1980; Lavergne and Trissl 1995; Kramer et al. 2004), the influence of connectivity between PSII units on the other processes has also been documented, e.g., through measurements on thermoluminescence (Tyystjärvi et al. 2009). The sigmoidicity of chlorophyll fluorescence induction has been found in control samples, i.e., those not treated with DCMU (Strasser and Stirbet 2001; Mehta et al. 2010, 2011). The phenomenon of connectivity is associated with excitation energy transfer between antenna complexes. They can be organized in different ways and they can create large domains, which probably enables the migration of excitation energy (Trissl and Lavergne 1995). Lambrev et al. (2011) have shown that in isolated thylakoid membranes four or more PSII supercomplexes formed connected domains. On the other hand, the excitation energy transfer between different layers of thylakoid membranes was not confirmed. This result supports the data of Kirchhoff et al. (2004) who found that stacking or unstacking of PSII membranes does not influence the connectivity parameter. The phenomenon of connectivity has been associated with the theory of PSII heterogeneity. It has been thought that the sigmoidal fluorescence arises from PSII α-centers located in the grana possessing large light-harvesting complexes, which are connected enabling migration of excitons. On the other hand, PSII β-centers located in the stroma lamellae emit fluorescence with exponential rise; this was explained by their small antenna size with negligible connectivity (Melis and Homann 1976). This hypothesis was also challenged, even though it is clear that PSII antenna size heterogeneity exists (see e.g., Vredenberg 2008; Schansker et al. 2013).

Although our estimate of the PSII connectivity may be approximate, substantial differences in the sigmoidicity of the fluorescence induction curves, observed in the values of curvature and probability of connectivity, lead us to conclude that the organization of PSII units (antenna size heterogeneity) in shade leaves differs from the sun leaves of barley. Hence, we speculate that the lower exciton transfer efficiency in shade leaves in HL contributes to maintaining the redox poise of PSII acceptors at physiologically acceptable level, similar to the level observed in sun leaves. This can partially explain rather low photoinhibitory quenching that we observe in shade barley leaves. The connectivity among PSII units is still a subject of discussion and its existence needs to be verified in different plant species, since the published results are contradictory (see above). However, our results suggest a physiological role for PSII connectivity. Moreover, we have shown that if the concept of connected PSII units is correct, omission of connectivity can lead (in special cases) to misinterpretation of the JIP-test results, as well as of some of the results of PAM measurements, on chlorophyll fluorescence.

The results, presented in this paper, show that LL growth conditions indeed induce changes in the photosynthetic apparatus of barley leaves. However, as a grassland species, barley mostly lacks the ability to acclimate efficiently to LL conditions. In this respect, it is not at all surprising that it does not create shade leaves with typical structural and functional characteristics that have been well described in woody plants and some herbs (Lichtenthaler et al. 1981; Lichtenthaler 1985; Givnish 1988; Evans 1996; Lichtenthaler et al. 2007). In contrast to many studies in other species, the shade character of the barley leaf was not associated with major changes in absorption cross section, as indicated by the absence of changes in Chl*a*/Chl*b* ratio as well as in parameters derived from the polyphasic ChlF induction. On the other hand, the shade character was obviously associated with high individual leaf area, lower total Chl content per leaf area unit, and low CO_2_ assimilation rate at HL intensities. In shade leaves, the electron transport was substantially limited; it was associated with decreases in the number of electron carriers and with decreased rates of electron transport to PSI. We have observed a very low connectivity (*p* ~ 0.28) among PSII units in shade leaves, as compared to that in sun leaves (*p* ~ 0.51). As we have demonstrated by the “connected units” model, the low connectivity of shade leaves may be beneficial to keep the excitation pressure lower, at physiologically more acceptable levels under HL conditions; this may protect the photosynthetic units against photodamage. HL-exposed shade leaves seem to adjust quickly to changed light conditions, mainly by enhancing electron transport between PSII and PSI.

## Electronic supplementary material

Below is the link to the electronic supplementary material.

Supplementary material 1 (PDF 65 kb)

## Abbreviations

ChlF: Chlorophyll *a* fluorescence
Chl *a*: Chlorophyll *a*
Chl *b*: Chlorophyll *b*
Cyt *b6*/*f*: Cytochrome *b6*/*f*
HL: High light
LHC: Light-harvesting complex
LL: Low light
OJIP: Polyphasic fast chlorophyll fluorescence induction, where O is for minimal fluorescence, P for peak, and J and I are inflections between O and P
PAM: Pulse amplitude modulation
PAR: Photosynthetic active radiation
PQ: Plastoquinone
Q_A_, Q_B_: Electron acceptors of PSII; primary and secondary quinones
PSII: Photosystem II
PSI: Photosystem I
RC: Reaction center
