# Supplementary material for: Photosynthetic responses of sun- and shade-grown barley leaves to high light: is the lower PSII connectivity in shade leaves associated with protection against excess of light?
Source: Photosynth Res. 2014 Jan 21;119(3):339–54. doi: 10.1007/s11120-014-9969-8 (PMC3923118; doi:10.1007/s11120-014-9969-8)
Supplement: Supplementary file 1 — Supplementary material 1 (PDF 65 kb) [file 11120_2014_9969_MOESM1_ESM.pdf]

SUPPLEMENTARY MATERIAL

Suppl. Table 1 Light conditions during the period between the leaf appearance and the time when the full expansion of leaf was achieved

Light regime	PAR (µmol photons m^{-2})		Total PAR Input (mol photons m^{-2})
	Average	Maximum	
Sun	469	1092	140
Shade	58	214	40

The highest recorded value of photosynthetic active radiation (Maximum PAR), mean value of all records excluding dark period (Average PAR) and the total sum of incident PAR during flag leaf expansion (Total PAR input).

Suppl. Table 2. Values of specific fluxes and phenomenological energy fluxes derived from chlorophyll *a* fluorescence in penultimate leaves of spring barley leaves acclimated to different light levels.

	Sun		Shade	
	B	HL	B	HL
Specific energy fluxes (per active PSII reaction center)				
ABS/RC	2.22	2.30	2.58	2.80
	(3.11)	(2.90)	(3.10)	(3.30)
TR_o/RC	1.79	1.75	2.02	1.97
	(2.54)	(2.21)	(2.40)	(2.32)
ET_o/RC	1.13	1.00	1.12	1.04
	(1.60)	(1.26)	(1.33)	(1.23)
DI_o/RC	0.44	0.55	0.56	0.83
	(0.62)	(0.69)	(0.67)	(0.98)

Mean values from 4 replicates. The numbers in parentheses represent the values per reaction center for “connected model”, calculated from data for unconnected by multiplying (1+C), where C represents the connectivity parameter (curvature; table 2). Sun – full light; Shade – light level ~ 13 % of full light. B – measurements before high light protocol; HL – measurements after high light protocol and dark adaptation. ABS/RC – absorbed photon flux per reaction centre (RC); TRo/RC – trapping flux per RC, ETo/RC – electron transport per RC; DIo/RC – dissipation per RC. More detailed description and calculation in text.

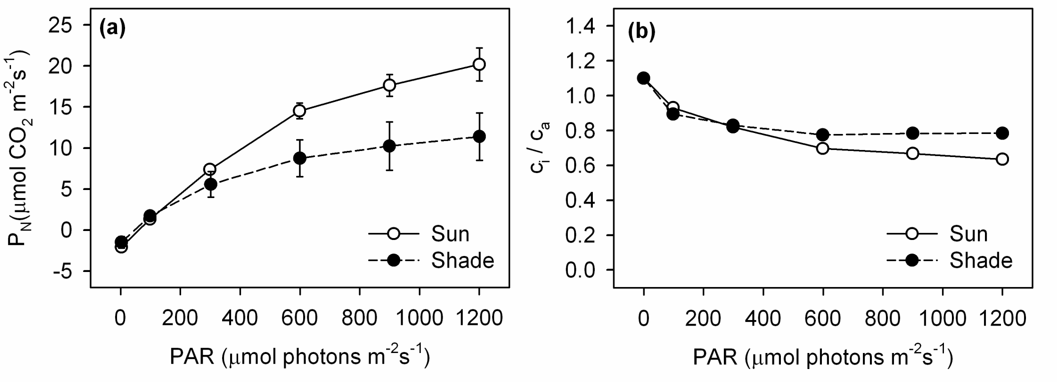

Suppl. Fig. 1 (**a**) Net photosynthetic rate (P_N), measured as CO_2 uptake in sun and shade leaves, plotted as a function of light intensity (0, 100, 300, 600, 900, 1,200 μmol photons m^{-2} s^{-1}, 15 min). (**b**) The ratio of the intercellular CO_2 concentration (c_i) to the concentration of CO_2 in air above the sample (c_a), both as a function of light intensity. Penultimate leaves of spring barley plants, acclimated to different light intensities during their entire growth period (○– sun leaf - 100 % of daylight, ● – shade leaf - 13 % of daylight). Mean values ± SE from 4 replicates. PAR on the abscissa stands for Photosynthetic Active Radiation.
